# Impact of agglomeration state of nano- and submicron sized gold particles on pulmonary inflammation

**DOI:** 10.1186/1743-8977-7-37

**Published:** 2010-12-02

**Authors:** Ilse Gosens, Jan Andries Post, Liset JJ de la Fonteyne, Eugene HJM Jansen, John W Geus, Flemming R Cassee, Wim H de Jong

**Affiliations:** 1 Centre for Environmental Health Research, National Institute for Public Health and the Environment, Bilthoven, the Netherlands; 2 Biomolecular Imaging, Faculty of Science, University of Utrecht, the Netherlands; 3 Laboratory for Health Protection Research, National Institute for Public Health and the Environment, Bilthoven, the Netherlands

## Abstract

**Background:**

Nanoparticle (NP) toxicity testing comes with many challenges. Characterization of the test substance is of crucial importance and in the case of NPs, agglomeration/aggregation state in physiological media needs to be considered. In this study, we have addressed the effect of agglomerated versus single particle suspensions of nano- and submicron sized gold on the inflammatory response in the lung. Rats were exposed to a single dose of 1.6 mg/kg body weight (bw) of spherical gold particles with geometric diameters of 50 nm or 250 nm diluted either by ultrapure water or by adding phosphate buffered saline (PBS). A single dose of 1.6 mg/kg bw DQ12 quartz was used as a positive control for pulmonary inflammation. Extensive characterization of the particle suspensions has been performed by determining the zetapotential, pH, gold concentration and particle size distribution. Primary particle size and particle purity has been verified using transmission electron microscopy (TEM) techniques. Pulmonary inflammation (total cell number, differential cell count and pro-inflammatory cytokines), cell damage (total protein and albumin) and cytotoxicity (alkaline phosphatase and lactate dehydrogenase) were determined in bronchoalveolar lavage fluid (BALF) and acute systemic effects in blood (total cell number, differential cell counts, fibrinogen and C-reactive protein) 3 and 24 hours post exposure. Uptake of gold particles in alveolar macrophages has been determined by TEM.

**Results:**

Particles diluted in ultrapure water are well dispersed, while agglomerates are formed when diluting in PBS. The particle size of the 50 nm particles was confirmed, while the 250 nm particles appear to be 200 nm using tracking analysis and 210 nm using TEM. No major differences in pulmonary and systemic toxicity markers were observed after instillation of agglomerated versus single gold particles of different sizes. Both agglomerated as well as single nanoparticles were taken up by macrophages.

**Conclusion:**

Primary particle size, gold concentration and particle purity are important features to check, since these characteristics may deviate from the manufacturer's description. Suspensions of well dispersed 50 nm and 250 nm particles as well as their agglomerates produced very mild pulmonary inflammation at the same mass based dose. We conclude that single 50 nm gold particles do not pose a greater acute hazard than their agglomerates or slightly larger gold particles when using pulmonary inflammation as a marker for toxicity.

## Background

The small size and subsequent relative increase in surface area-to-volume ratios of nanoparticles (NPs) results in special and desired properties for which NPs are engineered. It is hypothesized that this also contributes to making nanoparticles chemically more reactive leading to unexpected and aberrant effects upon interaction with biological systems compared to sub-micron sized particles of the same material. In a number of studies, the toxicity of nanoparticles (NPs) has been related to their small size and therefore high surface area per unit mass as well as proportionally having more atoms on the surface available for chemical reactions [1-7].

When performing a toxicity study, characterization of the test substance is of crucial importance in order to understand the parameters that can be used to predict the hazard of NPs. Primary particle size, shape, surface area, surface chemistry, surface charge, solubility or dissolution rate, purity and agglomeration state can all influence NPs toxicity [8]. In this study, the focus is on agglomeration state of particles of two different sizes and how this can influence the biological response after administration into the lung. Agglomeration of particles is a basic process that results in a reduction of surface free energy by increasing their size and decreasing their surface area. Agglomeration of nanoparticles is due to adhesion of particles to each other by weak forces leading to (sub)micronsized entities. In contrast, nanoparticle aggregates are due to the formation of covalent or metallic bonds that cannot be easily disrupted. Both agglomeration and aggregation raise questions how to address the evaluation of safety of NPs when they are no longer in the nano range, but are present as larger entities [9]. It is not known whether agglomerated particles could become single particles again when introduced in a biological system, while several studies have shown that single particles have the ability to form agglomerates in a biological matrix [10,11]. From a toxicological perspective, it is important to determine how the human body deals with single nanoparticles compared to agglomerates. In the workplace, inhalation of engineered nanoparticles is a realistic exposure scenario during production and handling [12]. Nanoparticles can exist in the form of single (primary) particles as well as agglomerates or aggregates.

Also the primary size of a particle (nano- versus submicron sized) could result in a different biological response. It is currently thought that single nanoparticles could (also) pose a greater hazard compared to larger particles due to their ability to translocate across barriers [13,14] and possibly to by-pass the pulmonary immune system e.g. by less efficient macrophage uptake [15]. The impact of agglomeration state of particles on these effects is not extensively studied.

Colloidal gold particles of 50 nm and 250 nm with a citrate coating are chosen as model particles to study effects on pulmonary endpoints, since they can be synthesized with a narrow size distribution as stable suspensions. Toxic effects of gold nanoparticles have been observed *in vitro*. For example, 14-nm colloidal gold particles were found to cross the cell membrane of dermal fibroblasts in culture and accumulate into vacuoles. The presence of the particles is responsible for abnormal actin filaments and extracellular matrix constructs, thereby inducing adverse effects on cell viability [16]. Conflicting results have been obtained regarding the cytotoxicity of gold, ranging from non-cytotoxic for 15 nm particles and 3.5 +/- 0.7 nm particles capped with lysine and poly-L-lysine [17] and to cytotoxic for 1.2 and 1.4 nm particles [18]. Gold 2 nm nanoparticles functionalized with cationic groups were moderately toxic in contrast to particles with anionic groups that were not toxic [19]. Gold particles have also been used to determine the fate as a function of size after different exposure routes. To what extent the agglomeration state will influence this, is largely unknown. In most studies, particles have been administered in an agglomerated state or as a mix of single and agglomerated particles via intravenous injection, intratracheal instillation and inhalation. After inhalation of nanogold particles (with a primary size of 5-8 nm and present in the aerosol as agglomerates of 30-110 nm or at least smaller than 100 nm, respectively), small quantities of particles translocate from the lung to other organs [20,21]. After intravenous injection [22] and intratracheal instillation [23,24], nano-sized particles have the ability to reach more distal regions of the body compared to their larger counterparts.

In this study we determine to what extend the agglomeration state of 50 nm and 250 nm particles influences pulmonary effects in the rat. A single dose of 1.6 mg/kg body weight of spherical gold particles of either 50 nm or 250 nm is intratracheally instilled in the rat lung after diluting either by 1/10 volume of ultrapure water to obtain single particles or by 1/10 volume of 10× phosphate buffered saline (PBS) to obtain agglomerates. This method of delivery was chosen over the physiological route of exposure via inhalation based on the possibility to administer particle solutions containing single particles versus agglomerates, more exact dosing, reduction of costs as well as less complexity in exposure of the animals. Intratracheal instillation is a widely accepted alternative for delivery of particles to the lung [25,26].

## Results

### Characterization of particles

The 50 nm and 250 nm gold particles were custom prepared at a concentration of 2 mg/ml and have a citrate shell for stabilization. Diluting the 50 nm particle solution using ultrapure water did not result in a colour change and particle size distribution measurements using the Nanosight apparatus indicated a highest peak size (size distribution peak with most particles) and mean particle size of around 50 nm (table 1), indicating that the citrate shell remains stabilized. Diluting the 50 nm particles using 1 part 10× PBS and 9 parts 50 nm particle suspension to obtain a physiological solution resulted in a colour change from red to blue with the highest peak and mean particle size above 100 nm indicating the formation of agglomerates [22]. The 50 nm particles in this dilution remained in suspension for approximately 5 minutes. Then particles started to precipitate at the bottom of the tube with a clearer aqueous solution on top. The resulting cluster of gold particles could no longer be homogenized by sonication. This effect was not observed for any other particle dilution that has been prepared. Therefore, 50 nm particles were mixed with PBS immediately before instillation and particle size distribution was measured directly afterwards to avoid this phenomenon. The highest peak size and mean particle size of 250 nm particles in ultrapure water were similar as indicated by a single distribution peak determined in the Nanosight apparatus. Dilution by using 10× PBS resulted in larger agglomerates with a large size distribution (table 1).

**Table 1:** Particle size distribution and zetapotential

	highest peak size ± sd	mean size ± sd	zetapotential (mV) ± sd
50 nm gold single	54 ± 3	61 ± 3	-56 ± 0,42

50 nm gold agg	114 ± 73	199 ± 88	-54 ± 1,01

250 nm gold single	197.5 ± 7	197 ± 12	-53 ± 0,53

250 nm gold agg	762 ± 762	768 ± 766	-61 ± 0,98

Highest peak size and mean particle size in this distribution is determined by tracking analysis of Brownian motion. Zetapotential is determined by using the Zetasizer instrument.

All solutions were electrically stabilized based on zetapotentials (between -40 and -60 mV) for single particles solutions as well as agglomerates (table 1). The pH measurement of the 50 nm and 250 nm gold particle solutions as well as the control solution indicated that they are in the physiological range of pH 6.4-7.0.

According to the supplier, the concentration of the gold particles suspensions should be 2 mg/ml. ICP-MS measurements revealed a lower concentration of 0.9 ± 0.02 mg/ml for 50 nm particles and 1.1 ± 0.02 mg/ml for 250 nm particles. The administered dose was 405 μg/rat equivalent to 1.6 mg/kg body weight (bw).

**Figure 1:**
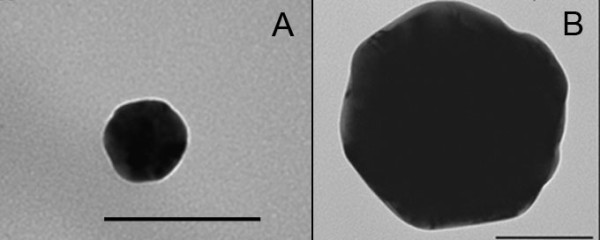
**TEM of single 50 and 250 nm particles**. A) Primary particle size of 50 nm gold particles was confirmed. B) The average primary particle size 250 nm gold particle was assessed at 210 nm. Bars represent 100 nm.

Using the Nanosight apparatus (table 1) as well as TEM (figure 1A and 1B), the particle size of the 50 nm particles was confirmed at 50 nm, while the 250 nm particles appeared to be smaller, namely 200 nm by tracking analysis versus 210 nm by TEM.

**Figure 2:**
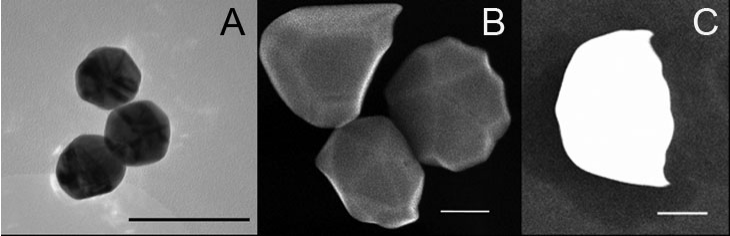
**TEM of 250 nm particles**. A) Some 250 nm gold particles consisted of multiple smaller units of about 50 nm. B) Secondary electron image of well faceted and grouped 250 nm particles. C) Example of a fractured 250 nm gold particle, secondary electron image. Bars represent 100 nm.

The gold particles of 50 nm appeared to be monocrystalline; the diffraction maxima and the lattice images pointed to pure Au (data not shown). Some gold particles of 250 nm consisted of two or three smaller not intimately connected particles. An example is shown in figure 2A. These composite 250 nm particles do not exhibit a monocrystalline diffraction pattern and a spherical or facetted shape as the 50 nm particles (data not shown). figure 2B represents three gold particles of about 250 nm. The secondary electron image demonstrated the individual particles to be well facetted, while other gold particles were fractured and had thus no symmetrical shape (figure 2C). The high-angle annular dark field (HAADF) images of both the 50 and the 250 nm particles showed the presence of areas containing carbon and oxygen on which some silicon containing areas were present, most likely in the form of SiO_2 _(data not shown). The carbon and oxygen may be due to the presence of citrates.

### Biological response

**Figure 3:**
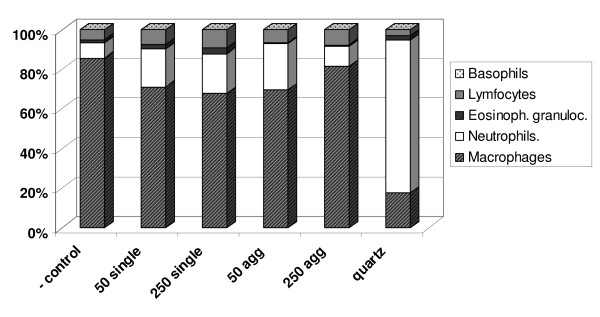
**Differential cell counts in BALF**. The percentage macrophages, neutrophils, eosinophilic granulocytes, lymphocytes and basophils were given per treatment group 24 hours after instillation (n = 6 for gold and n = 3 for quartz).

**Figure 4:**
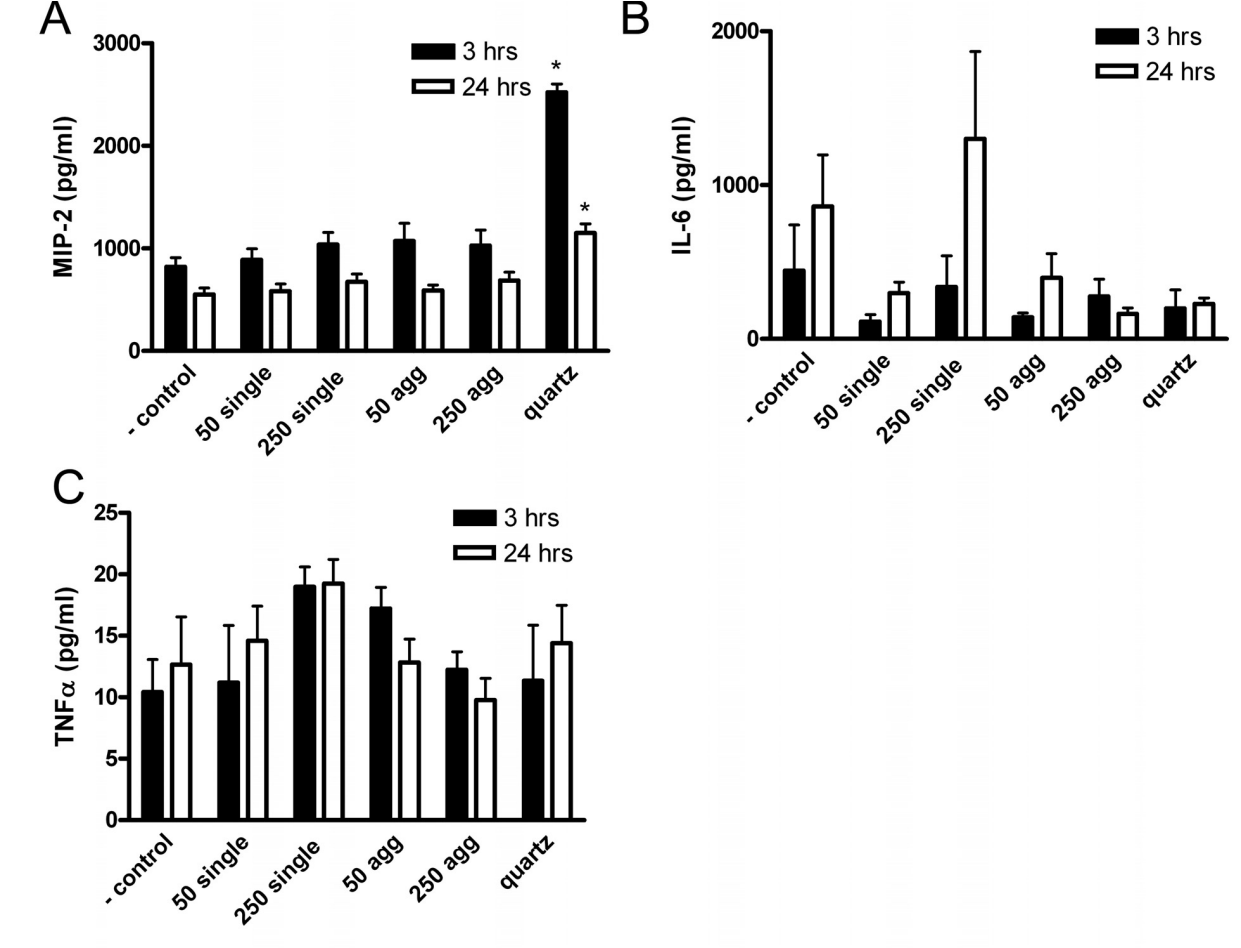
**Cytokine profile in BALF 3 and 24 hours after instillation**. A) MIP-2 levels are significantly increased after quartz instillation. * P < 0.05 (n = 6 for gold and n = 3 for quartz) B) No significant differences were found in IL-6 levels. A trend towards an increase is seen after instillation of 250 nm single gold particles. C) A trend towards an increase in TNF-α levels was found for 250 nm single gold particles and agglomerated 50 nm particles.

Three and 24 hours after instillation, several parameters were determined in the BALF. Single 250 nm particles induced a significant increase in the percentage of neutrophils after 24 hours (table 2). The percentage differential cell counts 24 hours after instillation showed that there is a shift towards a neutrophil influx at the expense of the percentage of macrophages compared to the negative control (figure 3), except for 250 nm agglomerates where the percentage in differential cell count did not change, albeit a trend towards an increase in total number of cells and absolute number of macrophages has been observed (table 2). This neutrophil influx was minor after gold particle treatment and much larger after quartz that is used as a reference material for inflammation (table 2 and figure 3). After quartz instillation, there was a significant increase in total cell numbers as well as macrophages after 3 hours and neutrophils after 24 hours. In addition, an increase in MIP-2 (figure 4A) and a tendency to an increase in MCP-1 levels (data not shown) was found, as expected [27]. TNF-α and IL-6 levels were around the detection limit. After instillation of 250 nm single gold particles, a trend towards an increase was found for IL-6 (figure 4B) and TNF-α levels (figure 4C). Agglomerated 50 nm particles resulted in increased levels of TNF-α after 3 hours, albeit not significant (figure 4C).

**Table 2:** Cell, macrophage and neutrophil concentration in BALF

	Total cell (10E6/ml)		Macrophage 10E6/ml)		Neutrophil (10E5/ml)	
**Treatment**	**3 hrs**	**24 hrs**	**3 hrs**	**24 hrs**	**3hrs**	**24 hrs**

- control	0.29 ± 0.05	0.72 ± 0.17	0.23 ± 0.06	0.61 ± 0.14	0.09 ± 0.02	0.57 ± 0.44

50 single	0.29 ± 0.04	0.71 ± 0.20	0.22 ± 0.04	0.55 ± 0.19	0.14 ± 0.07	0.99 ± 0.49

250 single	0.30 ± 0.11	0.74 ± 0.19	0.26 ± 0.11	0.49 ± 0.13	0.22 ± 0.16	1.5 ± 0.84*

50 agg	0.30 ± 0.10	0.54 ± 0.23	0.25 ± 0.10	0.39 ± 0.20	0.17 ± 0.13	1.0 ± 0.59

250 agg	0.36 ± 0.08	1.02 ± 0.45	0.30 ± 0.06	0.84 ± 0.42	0.19 ± 0.18	1.0 ± 0.76

quartz	0.54 ± 0.31*	2.16 ± 0.62*	0.47 ± 0.28*	0.32 ± 0.15	0.27 ± 0.14	17.2 ± 7.6*

Average concentration ± sd. Statistical significant differences P < 0.05 are indicated by *

**Figure 5:**
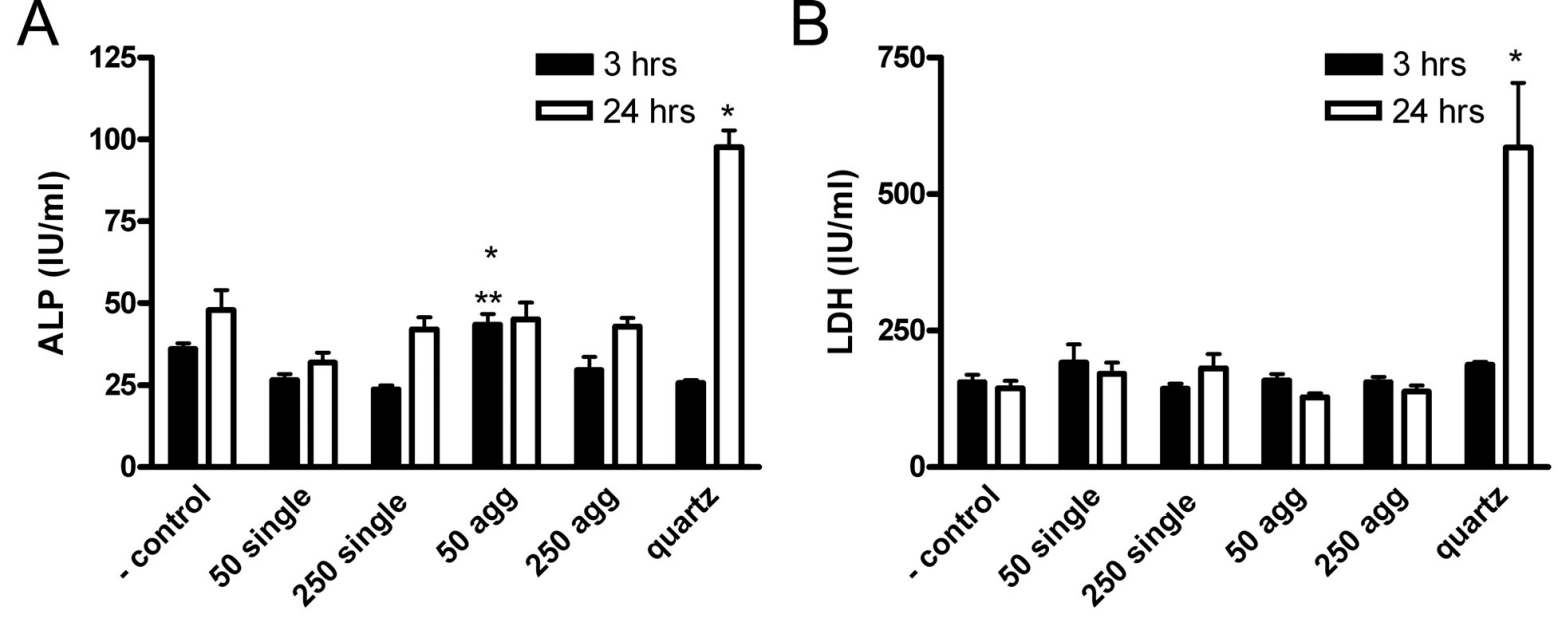
**Cell damage markers in BALF 3 and 24 hours after instillation**. A) Alkaline phosphatase (ALP) levels are significantly increased 3 hours after 50 nm gold agglomerates and 24 hours after quartz instillation. * P < 0.05 compared to negative control, ** P < 0.05 compared to 50 nm single gold particles. B) LDH levels are significantly increased 24 hours after quartz instillation (n = 6 for gold and n = 3 for quartz).

Analysis of damage markers total protein and albumin revealed no significant differences in the BALF after gold instillation (data not shown). The agglomerated 50 nm particles induced an increase in ALP (alkaline phosphatase) activity after 3 hours (figure 5A). Increased ALP activity in BALF has been associated with type II epithelial cell damage [28]. Quartz at 1.6 mg/kg bw resulted in cellular damage as can be seen by an increase in ALP (figure 5A) and lactate dehydrogenase (LDH) (figure 5B) after 24 hours.

**Figure 6:**
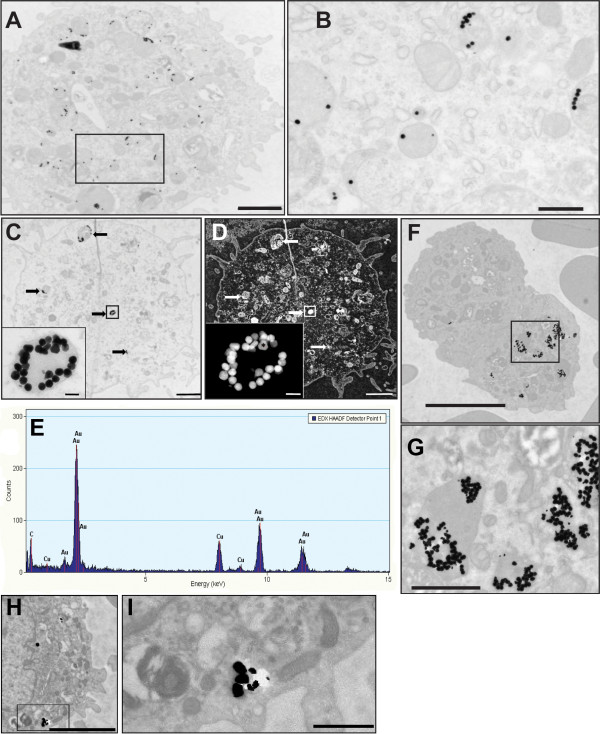
**TEM of macrophages in BALF 24 hours after instillation**. Uptake of 50 nm gold particles. Panels A-E concern instillation of gold particles in ultrapure water, panels F and G concern gold particles in PBS. A) Alveolar macrophage with multiple organelles containing 50 nm gold particles that were instilled as a well dispersed suspension containing single particles. B) Enlargement of the square in A, shows the presence of cellular structures containing one or more nanoparticles. C) Accumulation of agglomerates of nano-particles within organelles (arrows); the insert represents the vesicular structure containing over 25 nanoparticles in the black square. D) Same macrophage as shown in C now in HAADF mode. E) An EDX HAADF analysis has been performed on the nanoparticle indicated with an * in the insert and the presence of gold (Au) is confirmed. F) Agglomerated 50 nm gold particles were observed in the macrophages, although heterogeneity in cellular uptake is observed. G) Enlargement of the square in F. H) 250 nm gold particles were also found in the macrophages with an enlargement of the square in I). The bars represent: A, C, D: 2 μm, B: 500 nm, inserts C/D: 100 nm, F and H: 5 μm, G and I: 1 μm

Light microscopic analysis revealed dark coloured material inside macrophages of animals that were dosed with 50 nm and 250 nm particles, either agglomerated or as single particles. In control animals that did not receive any particles, no material was seen (Additional file 1, Figure S1). For both sizes of particles, 60-80 out of 100 macrophages contained this material. Transmission electron microscopy images showed that 50 nm and 250 nm particles were taken up by alveolar macrophages when the particles were administered either as single particles (figure 6A, 6B, 6C, 6D), as 50 nm agglomerated particles (figure 6F and 6G) or as 250 nm particles (figure 6H and 6I). Analysis of elements in HAADF mode confirmed that it were indeed gold particles (figure 6E).

**Figure 7:**
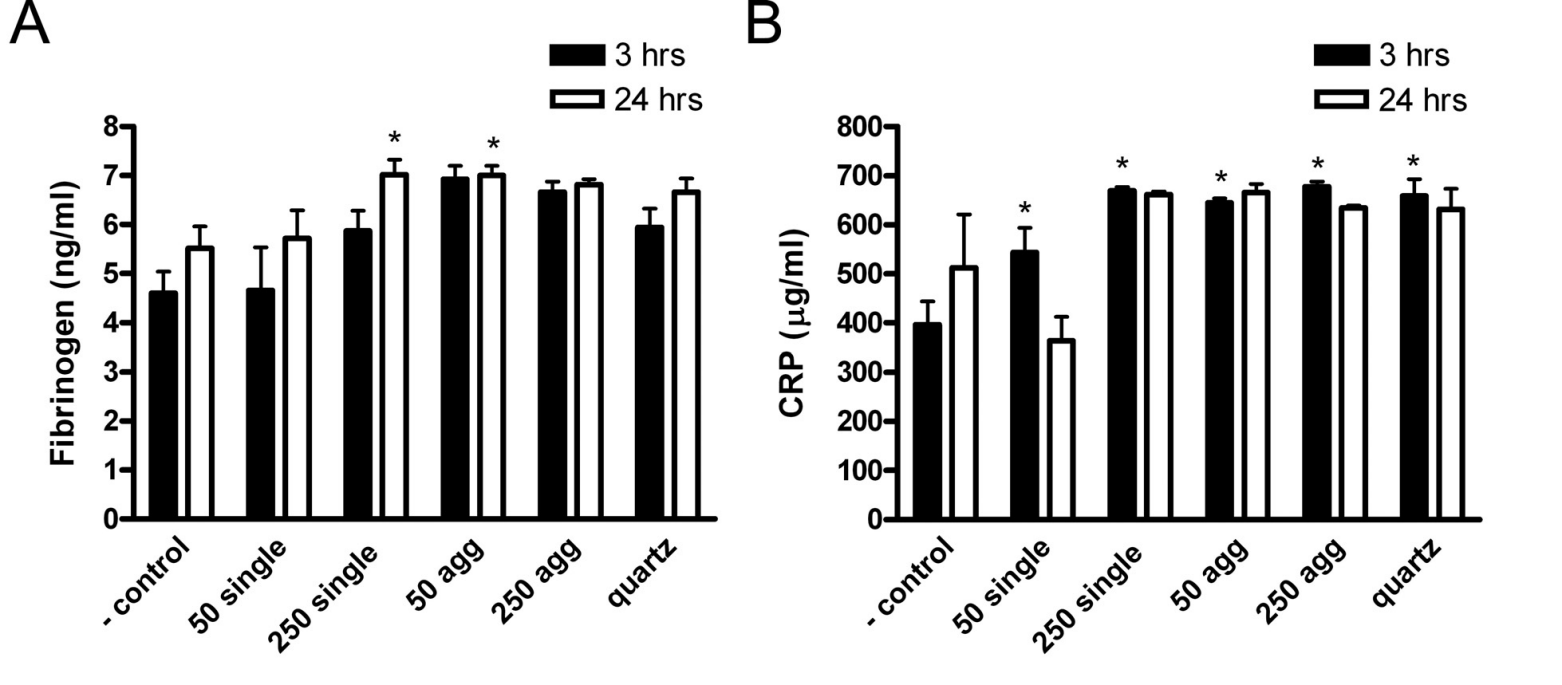
**Acute phase protein in serum 3 and 24 hours after instillation**. A) Fibrinogen levels are significantly increased after 250 nm single and 50 nm agglomerated particles. B) CRP levels are significantly increased 3 hours after gold and quartz treatment. * P < 0.05 compared to negative control (n = 6 for gold and n = 3 for quartz).

No significant differences in vWF and IL-6 levels in citrate plasma were found (data not shown). Single 250 nm and agglomerated 50 nm gold particles increased fibrinogen levels after 24 hours (figure 7A). After gold and quartz instillations CRP levels were elevated compared to the negative control after 3 hours (figure 7B). Upregulation of these acute phase proteins indicate a general response to tissue injury.

## Discussion

Size differences as well as the agglomeration state of citrate stabilized gold particles of 50 nm and 250 nm have been determined and the impact on the biological response in the lung has been established in this study. Size is one of the important characteristics for the deposition pattern and fate of particles in the body. Particle size affects the accessibility of target organs, the mode of cellular uptake, endocytosis and efficiency of particle processing in the endocytic pathway. Here, we show that 50 nm gold particles that were administered as a well dispersed single particle suspension or as an agglomerated suspension are taken up by macrophages. The particles end up in the cytoplasm inside vesicles and not in other structures. This has also been observed in another study in vivo study for agglomerated 5-8 nm gold particles [21]. In all cases, nanoparticles were surrounded by a membrane (figure 6), indicating that the uptake of the nanoparticles occurred by endo- or phagocytosis. Certain vesicular structures contain a single NP of 50 nm, whereas others contain more than just one. There is evidence for macrophage phagocytic function to be size dependent [5]. Alveolar macrophages phagocytise spherical particles of 1-2 μm most effectively and uptake has been seen up to 5 μm [29]. In vitro, particles of 300 nm are less well phagocytised compared to 5 μm [30]. Particle uptake in macrophages for nano-sized TiO_2 _has been estimated to be between 0.06 and 0.12% within 24 hours compared to micron sized particles. For the latter, 10% uptake is seen within the first hour [31] and more than 80% within 24 hours [15]. The set-up of this study did not allow quantification of the uptake. Although under the light microscope, the number of macrophages that were filled with material seemed comparable, it was more difficult to visualize macrophages containing 250 nm particles after 24 hours than 50 nm particles in TEM evaluation. This has also been seen before in our unpublished pilot study where we could visualize 250 nm particles in only a few macrophages.

The overall findings on biological parameters after gold instillation based on the pulmonary and blood markers are summarized in table 3. MIP-2 and MCP-1 were only affected by quartz and are therefore not mentioned in the table. TNF-α and IL-6 levels after quartz instillation were around detection levels as determined in two independent experiments using ELISA kits from different manufacturers. In literature, controversial results have been described with respect to increased TNF-α and IL-6 levels after quartz exposure. While some in vivo studies do show a significant increase [32], other studies only show an increase in vitro and not in vivo [33,34]. A NFκB inflammatory mechanism for DQ12 quartz that is partly TNF-α independent has been postulated [34].

**Table 3:** Overview significant changes and trends after gold instillation

	Local pulmonary effects						Systemic effects	
	**Inflammatory cells**			**Cytokines**		**Cell damage**	**Acute phase proteins**	
	**Cells**	**Macrophages**	**Neutrophils**	**IL-6**	**TNF-α**	**ALP**	**Fibrinogen**	**CRP**

50 nm single	-	-	↑	-	-	-	-	↑*

250 nm single	-	-	↑*	↑	↑	-	↑*	↑*

50 nm agg	-	-	↑	-	↑	↑*	↑*	↑*

250 nm agg	↑	↑	-	-	-	-	-	↑*

quartz	↑↑*	-	↑↑*	-	-	↑↑*	-	↑*

* indicates a statistically significant effect (P < 0.05).

When comparing 50 nm single versus 50 nm agglomerated particles, most changes on biological variables were found after instillation of agglomerated nanoparticles: a significant increase in ALP, fibrinogen and CRP is accompanied by a trend towards a neutrophil influx and an increase in TNF-α levels. Single 50 nm particles induced an increase in CRP levels and there was a trend towards an increase in neutrophils. Single 250 nm particles increased the number of neutrophils and the levels of fibrinogen and CRP. The response was accompanied by a trend towards an increase in TNF-α and IL-6. Agglomerated 250 nm particles only induced a significant increase in CRP. Moreover a (statistically not significant) increase in total cell number and the number of macrophages was noted. TNF-α is mainly produced by macrophages and stimulates phagocytosis. It also acts together with IL-6 (trend towards increase) by promoting inflammation by attracting neutrophils [35]. After increases in circulating TNF-α, the liver is stimulated to produce acute phase proteins like C-reactive protein (CRP) and fibrinogen. Most effects were found for single 250 nm particles, since all these parameters were affected. The 250 nm agglomerates, 50 nm agglomerates (that are the same mean size as the primary 250 nm particles) or 50 nm single particles did not affect these parameters to the same extent (table 1).

The hypothesis that single nanoparticles could be more toxic than larger counterparts either formed by agglomeration or larger primary size particles does not apply to the gold particles that were administered here in a single dose of 1.6 mg/kg bw to the lung. The least effects in this study were seen after administration of single 50 nm particles. At least two reasons have been hypothesized to explain why NPs could be more toxic: 1. there are more reactive atoms on the surface and more surface per unit mass. 2. nanoparticles that are small enough could exert quantum effects due to constraint chemical bonds that are more likely to be disrupted [36]. A recent systematic literature overview by Auffan *et al*. suggested that inorganic metal and metal oxide nanoparticles with a primary particle size below 20-30 nm are likely to show different chemical properties not seen in bulk material [37]. In the case of gold, catalytic activity has been observed for particles of smaller than 10 nm [38,39]. The nanoparticles of 50 nm used in this study are not likely to induce increased biological responses due to enhanced chemical reactivity compared to the submicron sized gold particles.

When suspended in PBS instead of ultrapure water, our study showed a 2-4 times increase in overall particle size. Particle agglomerate and aggregate formation in physiological media such as PBS has been observed for a number of different types of particles smaller than 100 nm resulting in entities that were 3-6 times larger [11]. These effects are influenced by pH, ionic strength, and different types of ions in aqueous suspensions [40]. Agglomerates are held together by weak forces such as van der Waals forces, electrostatic interactions or surface tension. After 5 minutes, 50 nm particles assembled to form large precipitates that could not be brought back into suspension using e.g. sonication. The gold nanoparticles have a negative surface charge as a result of the weakly bound citrate coating [41]. Gold colloids are vulnerable to agglomeration due to the compensation of the electrostatic repulsive force by the high ionic strength of phosphate buffered saline (PBS) [42]. In the present study we have carefully avoided this phenomenon by applying the suspensions to the animals and using the suspensions for chemical analyses immediately after preparation.

Extensive characterization of the particles revealed that the size of the 50 nm particles corresponds to the manufacturer's description. The 250 nm particles turn out to be significantly smaller and some are build-up from several nano-sized particles, whereas the 50 nm particles were monocrystalline. It turns out to be difficult to obtain gold particles with exactly the same characteristics with only the primary size as a variable. Due to differences in the production process, the 250 nm particles may have a different appearance and build-up compared to 50 nm particles as was shown by electron microscopy techniques.

## Conclusions

Both single and agglomerated 50 nm and 250 nm particles generate a mild inflammatory reaction after intratracheal instillation as indicated by small increases in inflammatory cells, pro-inflammatory cytokine production or acute phase protein expression. The effects are the least for single 50 nm gold particles. Both agglomerated as well as single nanoparticles were taken up by macrophages.

Extensive particle characterization reveals that primary particle size, concentration of the gold suspension and particle purity are important features to check, since these characteristics may deviate from the manufacturer's description. The hypothesis was that the lung might deal differently with agglomerated and single citrate stabilized gold nanoparticles of different sizes after intratracheal instillation, but there seem to be no major differences. We conclude that single 50 nm gold particles do not pose a greater acute hazard than their agglomerates or slightly larger gold particles when using pulmonary inflammation as a marker for toxicity.

## Methods

### Animals

Male WU Wistar-derived rats of 8 weeks of age and around 250 grams of body weight were obtained from Harlan, The Netherlands. Animals were bred under SPF conditions and kept barrier maintained during the experiment. Conventional feed (Special Diets Services) and tap water were provided ad libitum. Husbandry conditions were maintained according to all applicable provisions of the national law: Experiments on Animals Act. The experiment was approved by an independent ethical committee prior to the study.

### Experimental set-up

A single dose of 1.6 mg/kg bw in 0.5 ml of 50 nm or 250 nm gold particles was delivered in the rat lung by intratracheal instillation under isoflurane anaesthesia. The 50 nm and 250 nm gold suspensions (BBI International, UK) were custom prepared at 2 mg/ml. Solutions containing the same trace elements and reagents except for 50 and 250 nm gold particles (BBI International, UK) were used as a vehicle control (4.5 volumeparts control solution 50 nm and 4.5 parts 250 nm control solution and 1 part ultrapure water). Suspensions containing single 50 nm or 250 nm particles were prepared by diluting 9 volumeparts of 50 or 250 nm particles with 1 volumepart sterilized ultrapure water. Agglomerated (agg) suspensions were prepared by diluting 9 volume parts of particles with 1 volumepart sterilized 10× PBS. A single dose of 1.6 mg/kg bw quartz (DQ12, crystalline silica) in ultrapure water was used as a positive control. All solutions were sonicated for 30 seconds in an ultrasonic water bath prior to administration to the animals. Animals were sacrificed at 3 hours or 24 hours after administration of the particles, and bronchoalveolar lavage fluid (BALF) and blood were collected. Treatment groups for gold consisted of 6 animals and for quartz of 3 animals.

### Characterization of gold particles and quartz

Gold particles of 50 nm and 250 nm particles were purchased in sterile bottles and contained besides colloidal gold with a citrate shell, trace elements of substances used during synthesis. Endotoxin levels of the gold solutions of 50 and 250 nm were determined in a LAL assay and there were no detectable levels. The pH of the 50 and 250 nm solutions and control solutions were measured using indicator strips in the range of pH 1-10 and 6.4-8.0 (Merck). The zetapotential (Zetasizer, Malvern Instruments, UK) was determined in a triplicate measurement of a 20 μg/ml sample as a 10% dilution with ultrapure water or 10× PBS. The samples were first diluted with either ultrapure water or 10× PBS and then further diluted to the desired concentration using ultrapure water. Prior to preparing the quartz solutions, DQ12 was baked at 220°C for 3 hrs to inactivate possible endotoxin on the particle surface.

The concentration of gold in the commercial available solutions was determined by Inductively Coupled Plasma-Mass Spectrometry (ICP-MS) by MiPlaza Materials Analysis, Philips, Eindhoven, the Netherlands. Three independent samples were digested with aqua regia in a heating block system. The size distribution of the 50 nm and 250 nm gold particles directly after preparing the suspensions for intratracheal instillation were determined in 6 separate measurements using tracking analysis of Brownian motion with a laser illuminated microscopical technique (LM20, NanoSight Ltd, UK). The size of the 250 nm agglomerates were in the maximal range of the measurement technique resulting in three representative measurements.

### Transmission electron microscopy (TEM) analysis

A Tecnai 20F electron microscope equipped with a field-emission gun operated at 200 kV was employed to investigate the structure and the chemical composition of the gold particles with diameters of 50 and 250 nm. Samples were prepared by putting a drop of the suspension of the gold particles on a holey carbon film applied on a copper grid placed on filter paper. The gold particles were present well dispersed on the carbon film. Images were taken using conventional transmission electron microscopy (TEM) and scanning transmission electron microscopy (STEM) with a high-angle annular dark-field (HAADF) [43] and a secondary electron detector. STEM enabled us to execute elemental analysis by energy-dispersive X-ray (EDX) analysis at predefined spots. To assess the crystallographic structure of the gold particles, selected area electron diffraction and lattice imaging were performed.

TEM analysis of particles in lung cells in the BALF was performed by pooling cells per exposure group and by fixation in 2% glutaraldehyde in cacodylatebuffer (pH 7.2-7.2), supplemented with 0.025 mM CaCl_2 _and 0.05 mM MgCl_2_. The cells were embedded in gelatin, which was allowed to solidify to obtain "cell-tissue-blocks". The blocks were fixed in 4% paraformaldehyde (PFA), and postfixed with osmium tetroxide (OsO4) and potassium ferrocyanide (KFeCN), dehydrated with the use of ethanol and embedded in Epon. Ultrathin sections were made, post stained with lead citrate and uranylacetate and examined in a FEI Tecnai 12. Of the 24 hr groups at least three sections out of 12 sections were analyzed.

### Biological effect markers

At 3 and 24 hours after intratracheal administration, rats were anesthetized via i.p. injection with a mixture of Ketamine/Rompun and sacrificed by exsanguination via the abdominal aorta. The lungs were perfused with saline to remove all the blood in the tissue. After ligation of the left bronchus, the right lung was lavaged (three in-and-out lavages with the same fluid) with a volume of saline corresponding to 27 ml/kg of body weight at 37° to obtain BALF.

BALF was analyzed for the following parameters: total cell number (Coulter counter), differential cell count of 400 cells (cytospins), monocyte chemotactic protein -1 (MCP-1, Invitrogen), tumor necrosis factor α (TNF-α, Arcus Biologicals and eBiosciences), interleukin-6 (IL-6, Demeditec Diagnostics and eBiosciences) and macrophage inflammatory protein -2 (MIP-2, Arcus Biologicals). LDH, ALP, albumin and total protein were measured using an autoanalyser (LX20-Pro, Beckman-Coulter, Woerden, the Netherlands) using kits from the same manufacturer.

EDTA blood was used to determine the total number of cells and differential cell count. In citrate plasma, protein levels of van Willebrand factor (vWF, American Diagnostica), fibrinogen (Genway) and C-reactive protein (CRP, Helica Biosystems) were determined.

### Statistics

Data were analyzed by analysis of variance (ANOVA single factor) and where appropriate by a Bonferroni post-hoc analysis (Graphpad Prism). Differential cell count data are not normally distributed; therefore, the Kruskal-Wallis nonparametric test was used. Statistical significance is indicated with a * (P value < 0.05). In all graphs, error bars represent the standard deviation of the mean.

## Competing interests

The authors declare that they have no competing interests.

## Authors' contributions

IG designed the study. IG, JAP, LJJF and JWG carried out the study. IG collected, analyzed, interpreted data and drafted the manuscript. JAP, JWG, and EHJMJ generated and interpreted data. WHdeJ and FRC contributed to the study design and writing of the manuscript. All authors read and approved the final manuscript.

## Supplementary Material

Additional file 1 **Figure S1: Light microscopic images of particulate material in macrophages**. A) No particulate material is found inside macrophages from BALF in animals receiving the vehicle control. B and C) Animals received 50 nm gold particles. Black particulate material is seen in the cytoplasm (blue) and not in the nucleus (purple) of macrophages. D) Black particulate material is observed in macrophages of animals that received 250 nm gold particles. Bars represent 12 μm. Click here for file
