# Supplementary material for: Impact of agglomeration state of nano- and submicron sized gold particles on pulmonary inflammation
Source: Part Fibre Toxicol. 2010 Dec 2;7:37. doi: 10.1186/1743-8977-7-37 (PMC3014867; doi:10.1186/1743-8977-7-37)
Supplement: Additional file 1 — Figure S1: Light microscopic images of particulate material in macrophages. A) No particulate material is found inside macrophages from BALF in animals receiving the vehicle control. B and C) Animals received 50 nm gold particles. Black particulate material is seen in the cytoplasm (blue) and not in the nucleus (purple) of macrophages. D) Black particulate material is observed in macrophages of animals that received 250 nm gold particles. Bars represent 12 μm. [file 1743-8977-7-37-S1.DOC]

Additional file 1


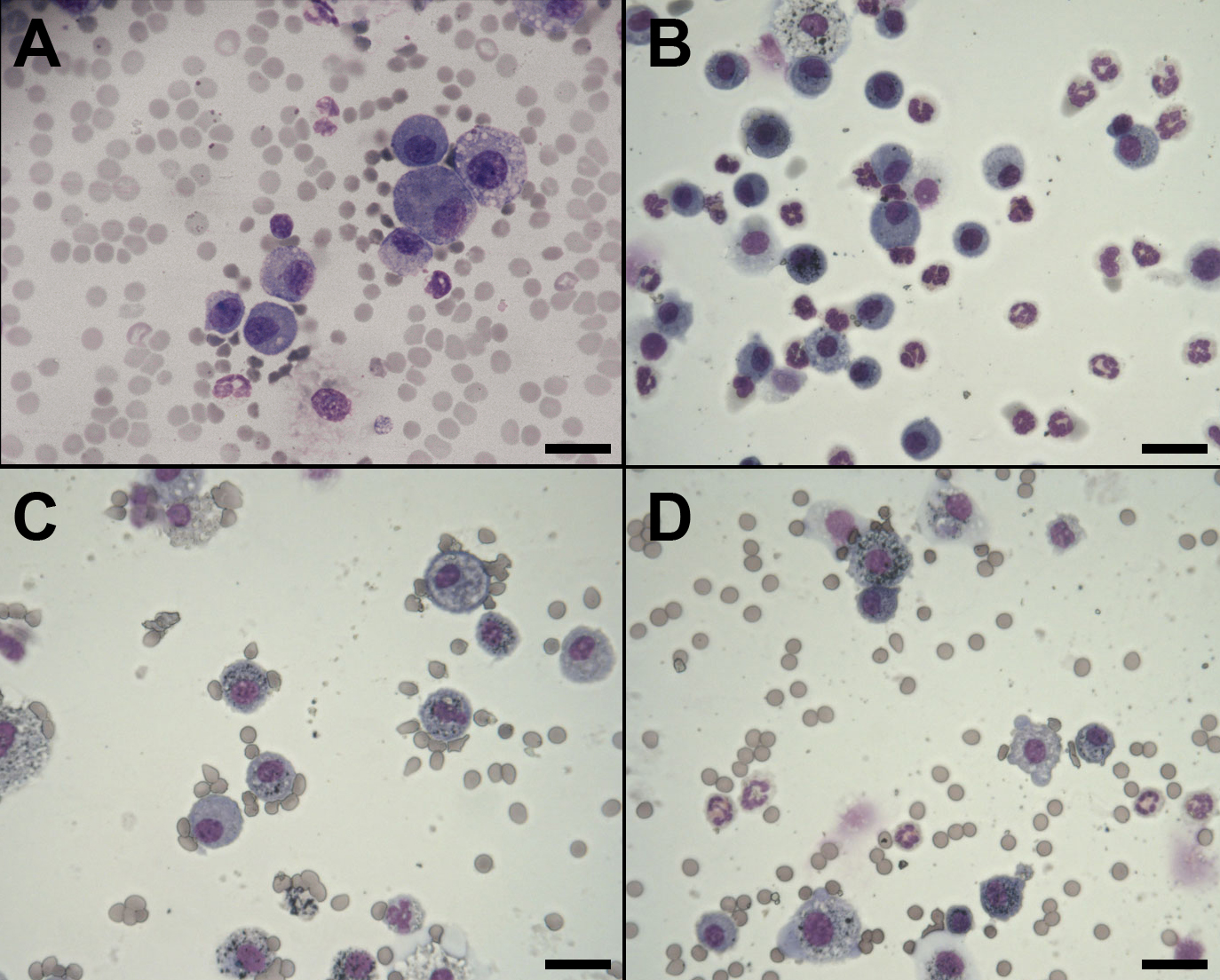


**Additional file, Figure S1 Light microscopic images of particulate material in macrophages**

A) No particulate material is found inside macrophages from BALF in animals receiving the vehicle control. B and C) Animals received 50 nm gold particles. Black particulate material is seen in the cytoplasm (blue) and not in the nucleus (purple) of macrophages. D) Black particulate material is observed in macrophages of animals that received 250 nm gold particles. Bars represent 12 µm.
